# Automated FRAP microscopy for high‐throughput analysis of protein dynamics in chromatin organization and transcription

**DOI:** 10.1002/2211-5463.70161

**Published:** 2025-12-06

**Authors:** Selçuk Yavuz, Bart Geverts, Johan A. Slotman, Andrea Sacchetti, Stefan Prekovic, Martin E. van Royen, Adriaan B. Houtsmuller

**Affiliations:** ^1^ Department of Pathology Erasmus University Medical Center Rotterdam The Netherlands; ^2^ Erasmus Optical Imaging Center Erasmus University Medical Center Rotterdam The Netherlands; ^3^ Center for Molecular Medicine UMC Utrecht The Netherlands

**Keywords:** androgen receptor, automated microscopy, cohesin, dynamic, FRAP, transcription

## Abstract

Fluorescence recovery after photobleaching (FRAP) is a quantitative technique to study the dynamics of fluorescently tagged proteins in living cells. Current FRAP workflows are limited in throughput because of the requirement for human interaction. Here, we present RoboMic, a fully automated confocal microscopy platform for high‐throughput imaging assays such as FRAP. We demonstrate its capabilities using two complementary approaches: sequential FRAP (sFRAP) and a novel parallel FRAP (pFRAP). The latter enables simultaneous photobleaching and monitoring of multiple cells within one imaging cycle, increasing throughput by approximately five‐ to 10‐fold while maintaining spatiotemporal resolution. The protocol consists of microscope control software for automated, AI‐based selection and segmentation of cell nuclei, sub‐nuclear ROI definition, photobleaching, and time‐lapse imaging. As proof of concept, we examined the nuclear dynamics of the androgen receptor and the cohesin complex under diverse conditions, demonstrating that RoboMic generates robust and reproducible data. In a single session, the platform yields hundreds of FRAP measurements, thereby increasing statistical power and scalability for large‐scale studies of protein mobility. While we focus here on FRAP, RoboMic can be readily applied to a wide range of quantitative functional imaging assays.

## Graphical abstract

RoboMic is an automated confocal microscopy pipeline for high‐throughput functional imaging in living cells. Demonstrated with fluorescence recovery after photobleaching (FRAP), it integrates AI‐driven nuclear segmentation, ROI selection, bleaching, and analysis. RoboMic increases throughput up to 10‐fold over manual FRAP and provides robust, reproducible measurements of protein dynamics, exemplified as proof of concept with androgen receptor variants and the cohesin complex.

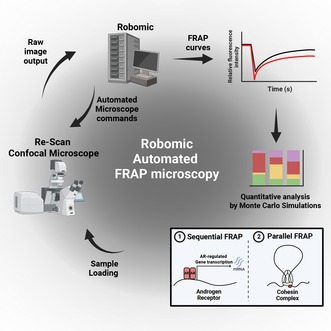

**Abbreviations**
AI: artificial intelligence
AR: androgen receptor
EGFP: enhanced green fluorescent protein
FRAP: fluorescent recovery after photobleaching
gRNA: guide RNA
OH‐F: hydroxyflutamide
pFRAP: parallel fluorescent recovery after photobleaching
RCM: ReScan confocal microscope
ROI: region of interest
sFRAP: sequential fluorescent recovery after photobleaching
SMC1A: structural maintenance of chromosomes protein 1A
SMC3: structural maintenance of chromosomes protein 3
STAG1: stromal antigen 1
STAG2: stromal antigen 2
WAPL: Wings apart‐like protein homolog

The investigation of how proteins move, interact, and bind within living cells is crucial for providing a better understanding of the mechanisms underlying their biological function(s). Several techniques exist to study protein dynamics, each with unique strengths and limitations. Among these, fluorescence recovery after photobleaching (FRAP) is a widely employed method that enables quantitative measurement of molecular diffusion in live‐cell environments [1, 2]. By selectively photobleaching a defined region of interest (ROI) inside a cell and monitoring the subsequent fluorescence recovery (either inside the ROI or outside), FRAP provides insight into protein mobility and interaction dynamics over spatial and temporal scales relevant to cellular processes [3].

FRAP workflows typically rely on substantial user interaction for cell selection, photobleaching, and data acquisition. This limits throughput and reduces statistical power, especially in heterogeneous populations or large‐scale screens. To address these challenges, we developed RoboMic, a fully automated, high‐throughput confocal microscopy software package designed to perform FRAP, in large‐scale experiments without time‐consuming intervention by operators. RoboMic supports both traditional sequential FRAP (sFRAP, Fig. 1B) and a novel parallel FRAP (pFRAP) mode (Fig. 1C). In sFRAP, individual nuclei are photobleached and monitored one at a time, providing high temporal resolution at the cost of throughput. In contrast, pFRAP allows multiple nuclei to be photobleached and imaged within a single cycle by grouping cells into batches and acquiring recovery data in parallel. This approach increases throughput approximately five‐ to 10‐fold compared to sFRAP, while maintaining sufficient temporal resolution to capture protein dynamics, depending on the microscope used and the recovery kinetics of the protein of interest. The RoboMic package integrates automated, artificial intelligence (AI)‐assisted cell segmentation, ROI selection, photobleaching, time‐lapse imaging, and data analysis using custom scripts.

**Fig. 1:**
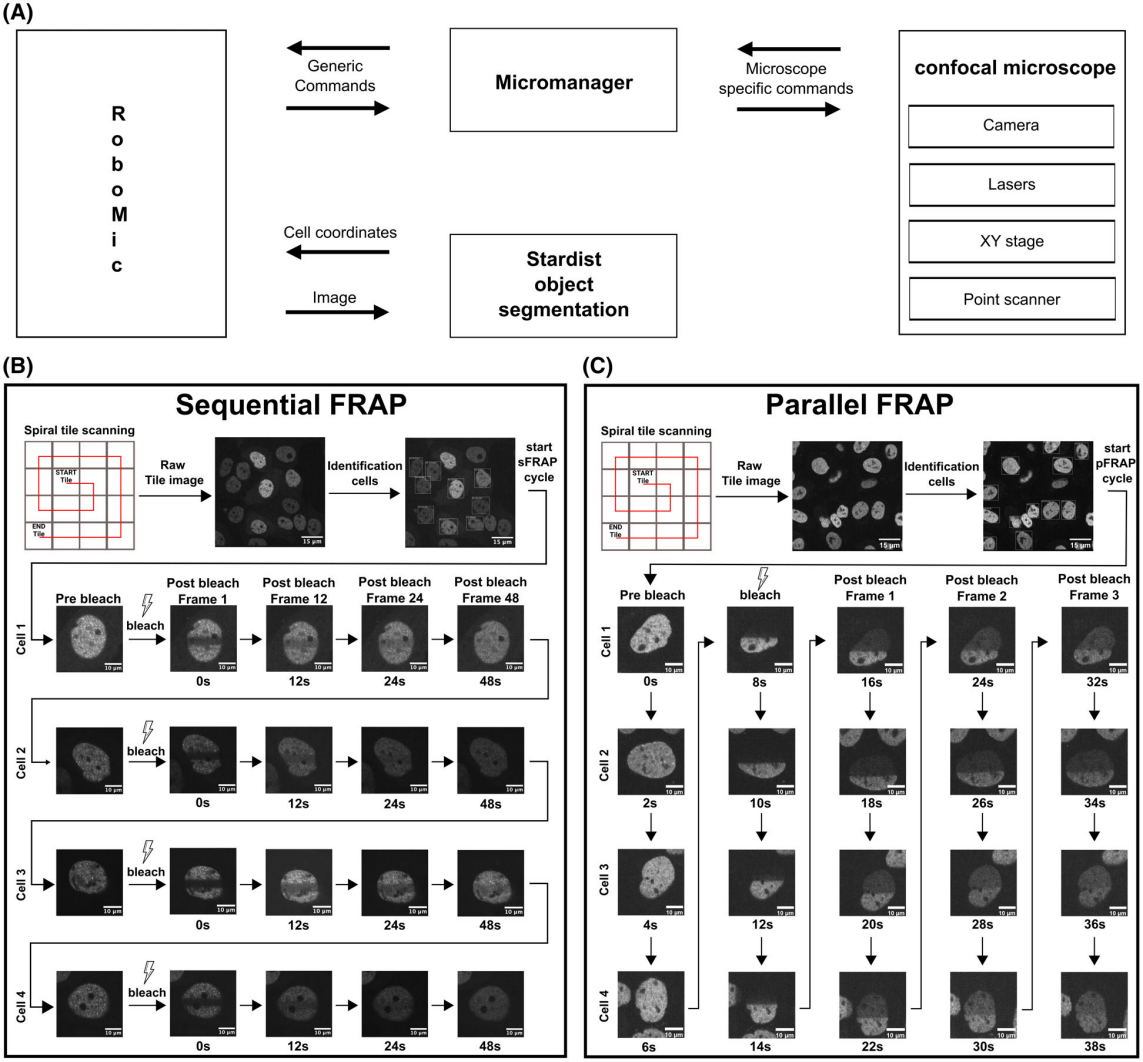
Overview of RoboMic and its application in sequential and parallel FRAP. (A) Schematic representation of the RoboMic system architecture. RoboMic interacts with the confocal microscope hardware through Micromanager, issuing generic commands that are translated into microscope‐specific instructions. Images acquired during tile scans are processed through StarDist‐based object segmentation to identify nuclei and extract cell coordinates for targeted FRAP. (B) Workflow and example images of Sequential FRAP (sFRAP). Following a tile scan and identification of cells, individual cells are photobleached and imaged one at a time over multiple postbleach frames, resulting in lower throughput but high temporal resolution. (C) Workflow and example images of Parallel FRAP (pFRAP). Multiple cells are photobleached and imaged in parallel within a single FRAP cycle, significantly increasing throughput by capturing recovery curves for several cells simultaneously. Scale bars = 10 μm.

To demonstrate the applicability of RoboMic, we first applied sFRAP to fluorescently labeled variants of the androgen receptor (AR). This nuclear hormone receptor regulates gene expression in response to androgen signaling and plays a central role in prostate cancer growth. Its nuclear dynamics, including DNA binding, are strongly influenced by ligand availability and provide a well‐established system to assess receptor mobility and chromatin interactions [3, 4, 5, 6]. To establish the power of pFRAP, we investigated the cohesin complex, a ring‐shaped protein complex essential for chromatin organization, sister chromatid cohesion, and transcriptional regulation. We labeled its core subunit SMC1A with EGFP and monitored the fluorescent recovery in multiple nuclei simultaneously. This significantly increased throughput compared to sFRAP. The mobility of SMC1A‐EGFP reflects changes in cohesin composition as a consequence of protein perturbations for cohesin cofactors, offering insights into how these different cohesin regulators influence chromatin‐binding dynamics in living cells.

Despite the widespread use of FRAP, existing approaches remain labor‐intensive, low‐throughput, and difficult to scale for systematic comparisons across proteins, cell lines, or conditions. RoboMic addresses these challenges by providing a modular and automated framework that combines the precision of FRAP with the scalability of high‐throughput screening, enabling reproducible, large‐scale measurements of protein dynamics in living cells.

## Materials

### Cell lines

Hep3B cells (HB‐8064; ATCC, Manassas, VA, USA) were cultured in Dulbecco's modified Eagle medium (DMEM) without phenol red (31053‐028; Gibco, Waltham, MA, USA) supplemented with 5% FCS (26140079; Gibco), 2 mm l‐glutamine (11679990; Lonza), penicillin/streptomycin (DE17‐602E; Lonza, Basel, Switzerland). EGFP‐labeled AR wild‐type, and the T878A anti‐androgen–insensitive mutant employed here were generated and characterized in earlier studies [4, 6]. The R585K DNA‐binding‐deficient mutant was generated by replacing the EYFP for an EGFP tag.

A549 cells (CCL‐185; ATCC) were cultured in DMEM/F12 (1 : 1) (1×) + Glutamax (10565018; Gibco) supplemented with 10% FCS (26140079; Gibco), 2 mm l‐glutamine (11679990; Lonza), penicillin/streptomycin (DE17‐602E; Lonza). Cells were passaged every 4 days with 0.05% Trypsin‐EDTA solution (25300054; Gibco).

### Generation of cohesin cofactor‐deficient SMC1A‐EGFP cell lines

The parental A549 SMC1A‐EGFP knock‐in cell line was established by Peralta *et al*. [7] and used to generate cohesin‐deficient subclones via CRISPR‐Cas9 with a single gRNA per gene. Guide RNAs were selected from the GeCKOv2 library to target *STAG1* (ACCACCTCAAATAATGTGAC), *STAG2* (AGTCCCACATGCTATCCACA), and *WAPL* (ACTACCCTTAGCACAAAATG). gRNAs were cloned into lentiCRISPRv2 as previously described [8], verified by Sanger sequencing, and packaged into lentivirus by transfecting HEK293T cells with pRC/CMV‐rev 1B, pHDM‐G, and pHDM‐Hgpm2 (1 : 1 : 1). For each construct, 17.5 μg CRISPR plasmid was combined with 10.5 μg packaging mix and 105 μL polyethylenimine (1 mg·mL^−1^), incubated for 15 min, and added to 3.5 × 10^6^ HEK293T cells plated the previous day in 10‐cm^2^ dishes. After overnight incubation, medium was replaced; supernatant was collected the next day and applied to A549 cells with polybrene (8 μg·mL^−1^). Cells were selected with puromycin (1.5 μg·mL^−1^) 48‐h postinfection.

## Methods

### Sample preparation

Hep3B stably expressing EGFP‐labeled AR variants and A549 SMC1A‐EGFP cell lines were seeded in 1.5H glass bottomed μ‐Slide 18 Well chambers (81816; Ibidi, Grafelfing, Germany). After overnight incubation at 37 °C, A549 cells were directly used for imaging while all Hep3B cell lines used in this study were additionally treated with medium containing 5% charcoal‐stripped FCS supplemented with 100 nm R1881 or 1 μm hydroxyflutamide (OH‐F) for 16 h at 37 °C prior imaging.

### Automated microscopy

Both sFRAP and pFRAP were performed on a ReScan Confocal (RCM) microscope unit (Confocal.NL, Amsterdam, the Netherlands) mounted on a Nikon Ti inverted microscope stand equipped with a stage top incubator providing 5% CO_2_ and 37 °C environmental conditions (Tokai Hit, Shizuoka‐ken, Japan), using a 60×/1.45 NA Plan APO TIRF oil immersion objective (Nikon, Tokyo, Japan), a 491 nm excitation laser line, and a BP 500–550 filter cube. The RoboMic package consists of scripts written in Java for both sFRAP and pFRAP modes (https://github.com/ErasmusOIC/robomic). Figure 1A illustrates the control architecture of the automated FRAP setup, where the RoboMic software communicates with the microscope through Micromanager, which translates generic commands from RoboMic into microscope‐specific instructions for the RCM confocal unit. Images captured by the microscope are fed into RoboMic and processed via Stardist object segmentation using the pretrained algorithm to extract cellular coordinates [9, 10]. These coordinates are then used to guide subsequent imaging and photobleaching steps, creating a fully automated feedback loop between software and hardware. This modular setup enables high‐throughput, reproducible measurements by integrating image analysis directly into the microscope control workflow. The minimal required laser power for monitoring of fluorescence was determined by pre‐experimental pilots to avoid significant decrease of fluorescent signal as a consequence of monitor bleaching.

To identify nuclei for both sFRAP and pFRAP procedures, an automated tile‐scanning routine was executed for each well, followed by nuclei selection (as illustrated in Fig. 1):

1. The microscope stage is positioned at the starting tile, corresponding approximately to the center of a well. The physical dimension of each tile is 102.3 × 102.3 micron.
2. A fluorescence image is acquired at this initial position.
3. Nuclei are detected and all identified nuclei are selected for imaging based on features such as intensity. The AI‐software package Stardist [10] has been incorporated in the detection step for stringent selection of nuclei based on its own algorithm. A Stardist score higher than 0.6 were set as threshold for nuclei selection.
4. Detected candidates are filtered based on threshold. For instance, a minimum distance between surrounding nuclei of bigger than 112 pixels were implemented to prevent FRAP measurements in two, too‐proximal neighboring cells. Also, a fluorescence intensity threshold was applied by selecting only the 20% most bright nuclei found in the tiles.
5. Adjacent tiles are scanned in an outward spiral pattern (Fig. 1B,C) centered on the starting tile to optimize coverage and to minimize the time needed required to move the stage from one to another position. Stage step size is 76.7 micron while maintaining a 25% overlap between tiles in order to prevent the chance of missing a cell during scanning.

This process is repeated until the desired number of nuclei is detected or a maximum tile limit is reached. Typically, nuclei are selected from 10 to 20 adjacent tile positions.

#### Sequential FRAP (sFRAP)

In the sequential FRAP protocol, one single nucleus is photobleached and monitored at a time before proceeding to the next nucleus (Fig. 1B):

1. The microscope stage moves to the starting position of the well, and tile scanning is performed as described above to select nuclei of interest.
2. For each selected nucleus:
  - The stage is moved to the center of the nucleus.
  - An image is acquired to refine the exact center of the nucleus (in case of cellular movements compared to the initial tile scan) and to calculate background fluorescence. Background fluorescence is used for background subtraction, which is required for FRAP measurements.
  - A binary mask of the nucleus is generated (by using the Stardist algorithm for nuclei segmentation).
  - The bleach strip is defined in the center of the nucleus based on the binary mask.
  - The fluorescence intensity measurements collected during the FRAP experiment are stored.
3. The sFRAP acquisition is performed in three, subsequently following phases by using a strip positioned in the middle of the binary mask:
  - Prebleach phase: A predefined number of images are acquired at defined intervals prior to bleaching and the intensities within the bleach strip are recorded for a predefined number of frames with a frame interval timing of 500 ms. A 20 μW 491 nm laser power was used for monitoring.
  - Bleach phase: A predefined number of bleaching cycles are applied within the selected bleach strip using 100% laser 491 nm power. Frame interval timing was set at 500 ms.
  - Postbleach phase: Fluorescence recovery in the bleached strip is recorded for a predefined number of frames with a frame interval timing of 500 ms. A 20 μW 491 nm laser power was used for monitoring.
4. During each FRAP measurement, background intensities were measured at 10 different locations outside the cell in ROIs of 16 × 16 pixels. This is used to subtract the background signal from the measurements, followed by normalizing the curves to prebleach [2, 3].
5. After each nucleus is processed, the FRAP data are saved in a text file.
6. Quantitative analysis of experimental FRAP curves is performed by using Monte Carlo simulations utilizing a minimal model as described by Geverts *et al*. [2].

### Case study

**Fig. 2:**
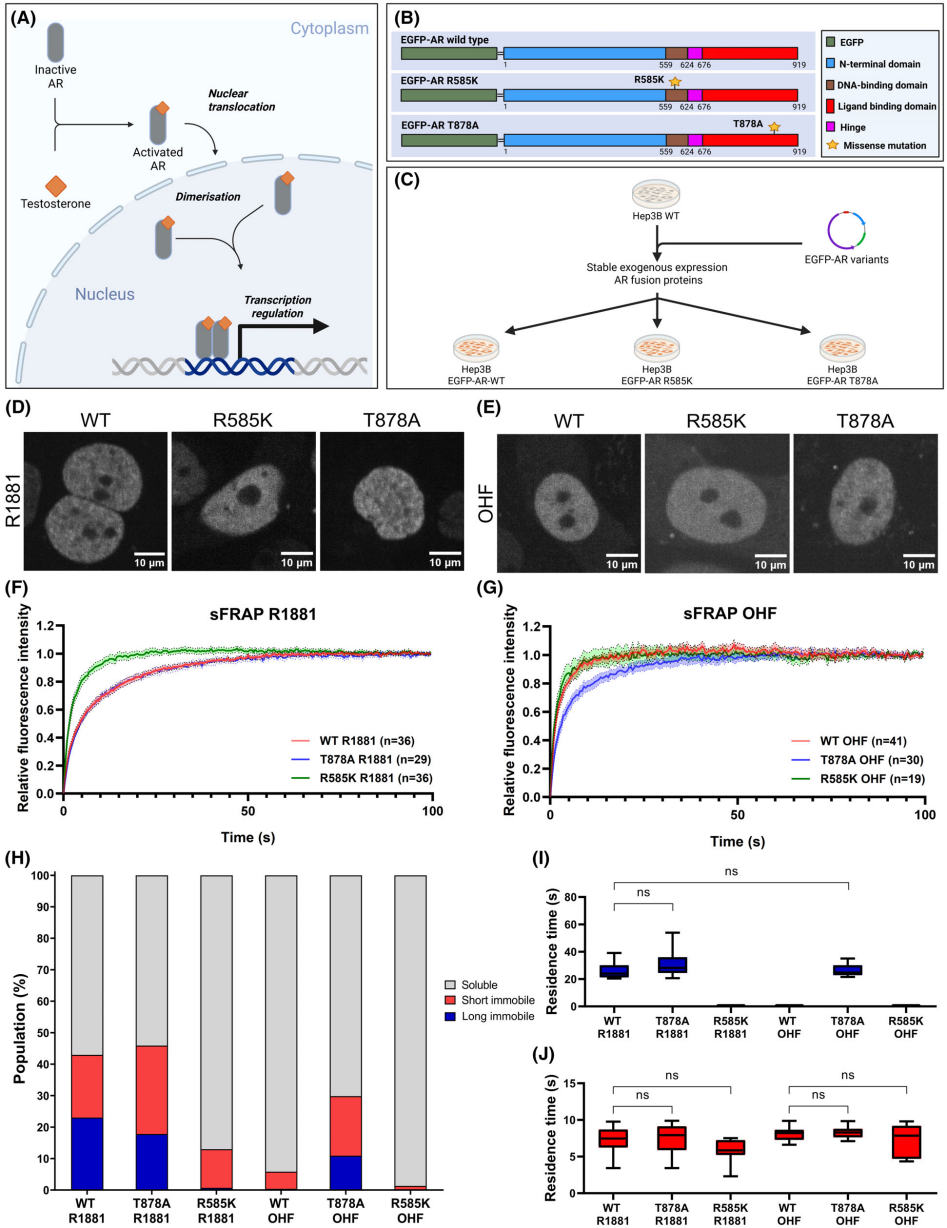
RoboMic enables high‐throughput measurements of androgen receptor mobility by sequential FRAP. (A) Schematic of androgen receptor (AR) activation and function. Upon testosterone binding, cytoplasmic AR undergoes nuclear translocation and dimerization in order to regulate transcription of AR‐target genes. (B) Domain structure of EGFP‐tagged AR variants used in this study, including wild‐type (WT), a DNA‐binding‐deficient mutant (R585K) and an anti‐androgen insensitive mutant (T877A). (C) Overview of the generation of Hep3B cell lines stably expressing EGFP‐tagged AR variants. (D, E) Representative confocal images showing nuclear localization of EGFP‐AR variants following treatment with the agonist R1881 (D) or the antagonist OH‐F (E). Scale bars = 10 μm. (F, G) FRAP recovery curves for each EGFP‐AR variant under R1881 (F) and OH‐F (G) treatment, measured using sequential FRAP (sFRAP). Curves represent average relative fluorescence intensity over time. (H) Population distribution of EGFP‐AR mobility states (soluble, short immobile, long immobile) under R1881 and OH‐F conditions, showing differences in binding dynamics between variants. (I, J) Quantification of average chromatin residence times under R1881 (I) and OH‐F (J) treatment for the short‐immobile and long‐immobile fractions. Box and Whiskers plots are generated from quantitative data derived from the best 10 fitting simulated curves. Error bars represent the range (minimum to maximum) of the data from the 10 best fits. Statistical significance was determined using a two‐sided Mann–Whitney–Wilcoxon test; ns, not significant.

To show the application of automated sFRAP by RoboMic, we determined the nuclear dynamics of wild‐type and mutant EGFP‐tagged androgen receptor (AR) variants in Hep3B cells (Fig. 2).

Cell lines stably expressing wild‐type AR, DNA‐binding‐deficient AR mutant R585K, and the T878A mutant that exhibits an antagonist‐to‐agonist switch in response to hydroxyflutamide (OH‐F) treatments were previously generated by genomic integrations of expression constructs (Fig. 2B,C) [3, 6]. Representative confocal images confirm nuclear localization of all variants upon treatment with the AR agonist R1881 (Fig. 2D) or AR antagonist OH‐F (Fig. 2E). Note that the agonist‐treated R585K mutant lacks AR condensates as a consequence of inefficient stable DNA‐binding while the antagonist‐treated T878A mutant is able to form AR condensates as described previously [4, 6, 11, 12, 13].

The average FRAP curves reveal that, unlike the DNA‐binding‐deficient R585K mutant, both wild‐type AR and the T878A mutant display the typical slow recovery upon R1881 stimulation, reflecting their stable chromatin interactions (Fig. 2F) [3, 4, 14]. Monte Carlo simulations of the experimental sFRAP curves confirm this, showing that under R1881 treatment the wild‐type AR and T877A mutant contain substantial immobile fractions (~ 23% and ~ 19%, respectively), whereas the R585K mutant is almost entirely soluble (~ 87%) with a small short‐immobile fraction (~ 12%) and no detectable long‐immobile population (Fig. 2H–J). In contrast, OH‐F treatment does not induce stable chromatin binding in wild‐type AR or the R585K mutant, both of which remain predominantly soluble (~ 94% and ~ 98%, respectively) and lack a long‐immobile fraction (Fig. 2G,H). Moreover, the OH‐F‐treated T877A mutant does exhibit a significant long‐immobile fraction (~ 13%). Residence time analysis further supports this conclusion: the long‐ and short‐immobile fractions of R1881‐treated wild‐type AR (26.3 ± 6.0 s and 7.2 ± 1.9 s, respectively) are similar to those of the R1881‐treated T877A mutant (31.2 ± 9.0 s and 7.4 ± 2.1 s), in stark contrast to the R1881‐treated R585K mutant which lacks a immobile fraction (Fig. 2I,J). Consistent with these findings, the OH‐F‐treated T877A mutant shows residence times for its immobile fractions (26.3 ± 4.4 s and 8.4 ± 0.9 s) comparable to those of R1881‐treated wild‐type AR and T877A (Fig. 2I,J) and consistent with previous reports [5, 14, 15].

Together, these results demonstrate the ability of automated sFRAP by RoboMic to quantitatively characterize distinct chromatin‐binding behaviors of AR variants in a ligand‐ and mutation‐dependent manner. Importantly, the observed differences between wild‐type AR, the DNA‐binding‐deficient R585K mutant, and the T878A mutant, which mediates an antagonist‐to‐agonist switch in response to certain anti‐androgens, are fully consistent with the established model of AR regulation and previous reports describing their molecular phenotypes [4, 5, 6, 11, 12]. These results not only confirm earlier findings but also demonstrate that the automated sFRAP approach is sensitive enough to detect subtle shifts in receptor mobility and chromatin residence times similar to manual FRAP. Moreover, using sFRAP for AR allowed for measuring approximately 40 cells per hour, which is approximately 3 times faster than manual FRAP of AR. More important, the RoboMic pipeline can perform as many experiments in one run over extended time (e.g., several consecutive days).

#### Parallel FRAP (pFRAP)

To further enhance throughput, a parallelized FRAP protocol is developed where multiple nuclei are imaged during one imaging cycle:

1. Spiral tile scanning and selection of nuclei are performed as in the sFRAP protocol.
2. The selected nuclei are grouped into batches (e.g., 3–5 nuclei), with the maximum available interval time per batch being determined by the overall execution speed of the microscope system, including confocal scanning, stage movement, and autofocus operations. Interval between each imaging cycles for all batches is set at 10 s (since SMC1A is a relative slow‐diffusing protein), but can be modified depending on the instrument processing speed and protein recovery kinetics (faster recovery requires shorter interval timing).
3. For each batch:
  • Each nucleus is sequentially centered (in case of cellular movements compared to initial tile scan) and imaged at full resolution.
  • Background fluorescence is calculated (used for background subtraction of FRAP measurements) and a binary mask is generated (by Stardist).
4. The pFRAP acquisition is performed in three, subsequently following phases by using a half‐cell ROI positioned in the middle of the binary mask:
  • Prebleach phase: All nuclei in the batch are imaged at defined intervals prior bleaching. Intensities within the mask are measured and stored for a predefined number of frames with a frame interval timing of 2 s. A 20 μW 491 nm laser power was used for monitoring.
  • Bleach phase: A predefined number of bleaching cycles are applied to each nucleus in sequence by using 100% 491 nm laser power. Frame interval timing was set at 2 s.
  • Postbleach phase: Recovery is monitored for a predefined number of frames for each nucleus with a frame interval timing of 2 s. A 20 μW 491 nm laser power was used for monitoring.
5. During each FRAP measurement, background intensities were measured at 10 different locations outside the cell in ROIs of 16 × 16 pixels. This is used to subtract the background signal from the measurements, followed by normalizing the curves to prebleach.
6. After each nucleus is processed, the FRAP data were saved in a txt. file.
7. Quantitative analysis of experimental FRAP curves is performed by using Monte Carlo simulations utilizing a minimal model as previously described in Geverts *et al*. [2].

Parallelization works only when the time‐lapse interval for imaging of a nucleus exceeds the total time needed to cycle through all nuclei in the batch. Under these conditions, postbleach recovery of each nucleus can be fully monitored without overlap from imaging other nuclei.

### Case study

**Fig. 3:**
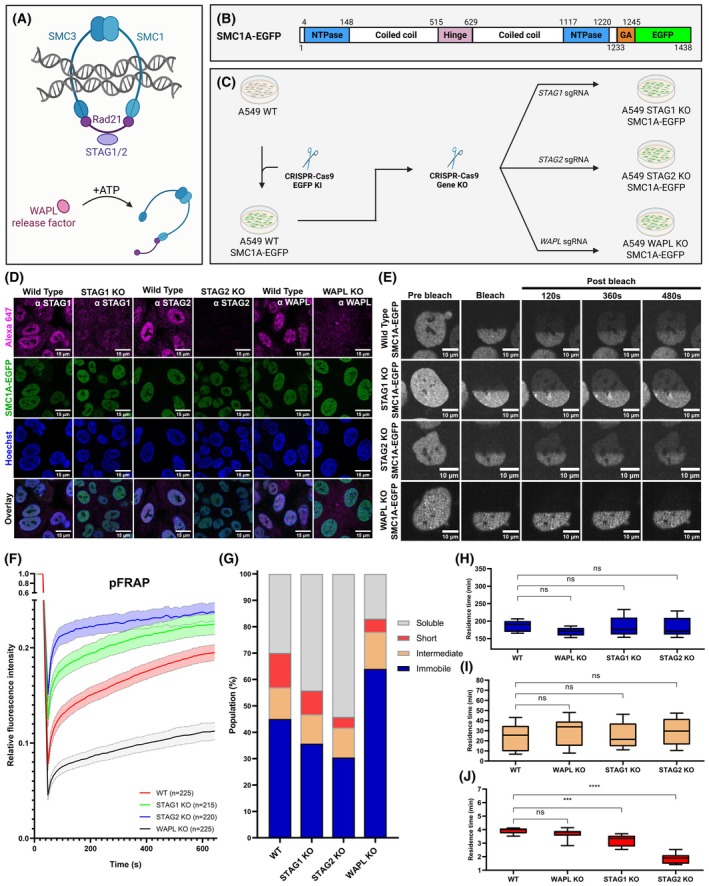
pFRAP reveals altered chromatin‐binding dynamics of SMC1A in cohesin regulator knockout A549 cell lines. (A) Schematic of the cohesin complex. The core ring consists of SMC1A, SMC3, and RAD21, supplemented by either STAG1 or STAG2 to form a DNA‐bound protein complex while WAPL promotes the release of cohesin from chromatin in an ATP‐dependent manner. (B) Domain structure of EGFP‐tagged SMC1A, showing functional regions including NTPase domains, coiled‐coil regions, hinge, a glycine–alanine (GA) spacer and an EGFP tag. (C) Generation of EGFP knock‐in and gene knockout A549 cell lines. CRISPR‐Cas9 was used to introduce EGFP at the endogenous SMC1A locus at the C terminus, followed by independent knockouts of STAG1, STAG2, or WAPL to generate isogenic SMC1A‐EGFP lines deficient for these key cohesin regulators. (D) Immunofluorescence images confirming loss of STAG1, STAG2, or WAPL in respective knockout lines expressing SMC1A‐EGFP. EGFP (green), antibody staining (magenta), and nuclear DNA (Hoechst, blue) are shown. (E) Representative pFRAP images of wild‐type and knockout SMC1A‐EGFP cells before bleaching, immediately after bleaching, and during fluorescence recovery. Scale bars = 10 μm. (F) Mean pFRAP recovery curves of SMC1A‐EGFP in wild‐type and knockout cell lines, showing distinct recovery kinetics across conditions. Shaded areas represent 95% CI. (G) Population distribution of SMC1A‐EGFP mobility states based on FRAP recovery dynamics, categorized as soluble, short‐binding, intermediate, or immobile. (H–J) Quantification of SMC1A‐EGFP chromatin residence times in the immobile (H), intermediate (I), and short‐binding (J) fractions across the wild‐type and knockout backgrounds. Box and Whiskers plots are generated from quantitative data derived from the best 10 fitting simulated curves. Error bars represent the range (minimum to maximum) of the data from the 10 best fits. Statistical significance was determined using a two‐sided Mann–Whitney–Wilcoxon test; ****P < 0.0001; ***P < 0.001; ns, not significant.

To show the application of pFRAP using RoboMic, we assessed the chromatin‐binding dynamics of endogenous cohesin complex member SMC1A‐EGFP in wild‐type A549 cells and in isogenic A549 knockout lines lacking key cohesin subunits. The cohesin complex plays a central role in genome organization by mediating sister chromatid cohesion, chromatin looping, and transcriptional regulation. Its activity depends on dynamic chromatin association and release, making it an ideal system to study long‐immobilized protein dynamics in living cells [16]. The complex itself is ring‐shaped and composed of the core subunits SMC1A, SMC3, and RAD21, together with one of the two variant STAG subunits, STAG1 or STAG2, while WAPL mediates its release from chromatin (Fig. 3A). As an experimental model, we generated an A549 cell line in which endogenous SMC1A was tagged with EGFP (Fig. 3B), allowing direct visualization and quantitative analysis of cohesin dynamics [7]. From this parental line, we established SMC1A‐EGFP sublines deficient in STAG1, STAG2, or WAPL using CRISPR‐Cas9‐mediated gene editing (Fig. 3C). Successful knockout of each cofactor was confirmed by immunofluorescence staining for STAG1, STAG2, and WAPL, showing loss of the respective protein signals in the corresponding cell lines, while nuclear localization of SMC1A‐EGFP was retained (Fig. 3D). Representative pFRAP image series (Fig. 3E) display fluorescence before bleaching, immediately after bleaching, and during recovery. These illustrate the effective photobleaching and recovery dynamics captured in both wild‐type and knockout conditions.

The mean FRAP recovery curves reveal that SMC1A‐EGFP is less mobile in WAPL‐deficient cells, showing slower recovery and therefore more stable chromatin association (Fig. 3F). Monte Carlo simulations confirm this, estimating an increased immobile fraction of about 65% in WAPL knockout cells compared to ~ 45% in wild‐type (Fig. 3G). In contrast, STAG1 and STAG2 knockout cells display faster recovery kinetics, consistent with reduced chromatin binding, which is reflected in their lower immobile fractions of ~ 35% and ~ 31%, respectively (Fig. 3F,G). While intermediate and short‐mobility fractions varied only slightly between conditions, the soluble fraction shifted markedly in the opposite direction of the immobile fraction. Specifically, the soluble pool decreased in WAPL‐deficient cells (~ 17%) compared to wild‐type (~ 30%), but increased in STAG1 (~ 44%) and STAG2 (~ 54%) knockouts (Fig. 3G). Residence time analysis further shows that the immobile and intermediate fractions did not differ significantly between conditions (Fig. 3H,I). However, the short fraction displayed altered binding kinetics: STAG1 (3.22 ± 0.44 min) and STAG2 (1.85 ± 0.37 min) knockouts both had shorter residence times compared to wild‐type (3.92 ± 0.20 min) (Fig. 3J). Together, these results indicate that WAPL loss stabilizes cohesin–chromatin interactions by increasing the immobile fraction of SMC1A, as reported previously by others [17, 18]. In contrast, depletion of either STAG1 or STAG2 reduces stable chromatin binding and shifts cohesin toward more dynamic states, especially for STAG2‐deficient cells, suggesting that both subunits contribute to maintaining chromatin residence of the core complex in different ways [19, 20]. The shorter residence times of the short‐mobile fraction in STAG1/2 knockouts further emphasize that cohesin complexes lacking these subunits are less stably engaged with chromatin, pointing to complementary, (partially) nonredundant roles of STAG1 and STAG2 in regulating the chromatin conformation by cohesin [19, 21].

In conclusion, these results demonstrate that RoboMic enables fully automated and parallelized pFRAP measurements pFRAP for SMC1A‐EGFP allows for measurements of approximately 24 cells per hour, which is approximately 5 times faster than sFRAP. More important, the RoboMic pipeline can perform as many experiments in one run over extended time (e.g., several consecutive days), providing a powerful platform to efficiently compare chromatin‐binding dynamics across multiple conditions in a time‐efficient manner.

## Tips and tricks

- RoboMic is a universally applicable software package for automated imaging assays on confocal microscopes, as long as these systems allow the usage of external software control packages.
- Spiral tiling pattern minimizes distances between cells to be imaged while covering sufficient area for nucleus detection.
- A mask‐based nuclear segmentation ensures consistency in ROI selection for FRAP.
- RoboMic can be also applied in FRAP approaches that require alternative bleaching region of interests, to study the protein dynamics in, for example, nuclear foci (spot‐FRAP) or in cytoplasmic organelles or structures. These approaches will require custom ROI selections for the proteins of interest.
- In pFRAP, batch size should be optimized based on the time required for stage movement between nuclei.
- In pFRAP, imaging intervals should be optimized based on the time it takes for a protein of interest to reach final recovery intensity.
- Batch size and imaging interval should be well balanced.
- Proteins showing relatively slow‐recovering fluorescent intensity allow for parallel analysis of multiple nuclei (pFRAP).
- Importantly, pFRAP procedures which involve very long postbleach monitoring requires additional correction for movement, which needs to be developed as add‐on for RoboMic.
- In addition to FRAP application, RoboMic can be also used for most other quantitative functional imaging assays.
- RoboMic can be customized for parallel measurements of multiple ROIs in the same nucleus.

## Conflict of interest

The authors declare no conflict of interest.

## Author contributions

SY, BG, MER, and ABH conceived and supervised the study; SY and SP generated CRISPR‐Cas9 modified A549 EGFP knock‐in and knockout cell lines; SY and AS sorted the cell lines used in this study by standard FACS procedures; BG conceptualized, developed, and implemented the RoboMic software package for autonomous microscopy with input from SY and ABH; BG performed FRAP experiments and the implementation of quantitative FRAP analysis by Monte Carlo modeling; BG acquired high‐resolution rescan images of cells; SY and BG prepared figures; SY wrote the manuscript with input of other co‐authors. All authors have read and approved the final manuscript.

## Data Availability

Raw imaging data can be provided upon request by the authors.

## References

[1] Houtsmuller AB , Rademakers S , Nigg AL , Hoogstraten D , Hoeijmakers JH and Vermeulen W (1999) Action of DNA repair endonuclease ERCC1/XPF in living cells. Science (1979) 284, 958–961. 10.1126/science.284.5416.958 10320375

[2] Geverts B , van Royen ME and Houtsmuller AB (2015) Analysis of biomolecular dynamics by FRAP and computer simulation, 109–133. 10.1007/978-1-4939-2080-8_7 25391797

[3] van Royen ME , Farla P , Mattern KA , Geverts B , Trapman J and Houtsmuller AB (2008) Fluorescence recovery after Photobleaching (FRAP) to study nuclear protein dynamics in living cells. In Methods in Molecular Biologypp. 363–385. Springer Protocols, New York. 10.1007/978-1-60327-461-6_20 18951195

[4] van Royen ME , van Cappellen WA , de Vos C , Houtsmuller AB and Trapman J (2012) Stepwise androgen receptor dimerization. J Cell Sci 25, 1970–1979. 10.1242/jcs.096792 22328501

[5] Van Royen ME , van Cappellen WA , Geverts B , Schmidt T , Houtsmuller AB and Schaaf MJM (2014) Androgen receptor complexes probe DNA for recognition sequences by short random interactions. J Cell Sci 127, 1406–1416. 24481814 10.1242/jcs.135228

[6] Farla P , Hersmus R , Trapman J and Houtsmuller AB (2005) Antiandrogens prevent stable DNA‐binding of the androgen receptor. J Cell Sci 118, 4187–4198. 16141232 10.1242/jcs.02546

[7] Mayayo‐Peralta I , Gregoricchio S , Schuurman K , Yavuz S , Zaalberg A , Kojic A , Abbott N , Geverts B , Beerthuijzen S , Siefert J *et al*. (2023) PAXIP1 and STAG2 converge to maintain 3D genome architecture and facilitate promoter/enhancer contacts to enable stress hormone‐dependent transcription. Nucleic Acids Res 51, 9576–9593. 37070193 10.1093/nar/gkad267 PMC10570044

[8] Shalem O , Sanjana NE , Hartenian E , Shi X , Scott DA , Mikkelson T , Heckl D , Ebert BL , Root DE , Doench JG *et al*. (2014) Genome‐scale CRISPR‐Cas9 knockout screening in human cells. Science 343, 84–87. 24336571 10.1126/science.1247005 PMC4089965

[9] Schmidt U , Weigert M , Broaddus C and Myers G (2018) Cell detection with star‐convex polygons. In Medical Image Computing and Computer Assisted Intervention – MICCAI 2018 ( Frangi Alejandro F and Schnabel JA , eds), pp. 265–273. Springer International Publishing, Cham.

[10] Weigert M and Schmidt U (2022) Nuclei instance segmentation and classification in histopathology images with Stardist. In 2022 IEEE International Symposium on Biomedical Imaging Challenges (ISBIC), pp. 1–4.

[11] Yavuz S , Kabbech H , van Staalduinen J , Linder S , van Cappellen WA , Nigg AL , Abraham TE , Slotman JA , Quevedo M , Poot RA *et al*. (2023) Compartmentalization of androgen receptors at endogenous genes in living cells. Nucleic Acids Res 51, 10992–11009. 37791849 10.1093/nar/gkad803 PMC10639085

[12] Yavuz S , Abraham TE , Houtsmuller AB and van Royen ME (2024) Phase separation mediated sub‐nuclear compartmentalization of androgen receptors. Cells 13, 1693. 39451211 10.3390/cells13201693 PMC11506798

[13] El Kharraz S , Dubois V , van Royen ME , Houtsmuller AB , Pavlova E , Atanassova N , Nguyen T , Voet A , Eerlings R , Handle F *et al*. (2021) The androgen receptor depends on ligand‐binding domain dimerization for transcriptional activation. EMBO Rep 22, e52764. 34661369 10.15252/embr.202152764 PMC8647150

[14] Farla P , Hersmus R , Geverts B , Mari PO , Nigg AL , Dubbink HJ , Trapman J and Houtsmuller AB (2004) The androgen receptor ligand‐binding domain stabilizes DNA binding in living cells. J Struct Biol 147, 50–61. 15109605 10.1016/j.jsb.2004.01.002

[15] Prekovic S , van Royen ME , Voet ARD , Geverts B , Houtman R , Melchers D , Zhang KYJ , Van den Broeck T , Smeets E , Spans L *et al*. (2016) The effect of F877L and T878A mutations on androgen receptor response to enzalutamide. Mol Cancer Ther 15, 1702–1712. 27196756 10.1158/1535-7163.MCT-15-0892

[16] Scott JS , Al Ayadi L , Epeslidou E , van Scheppingen RH , Mukha A , Kaaij LJT , Lutz C and Prekovic S (2025) Emerging roles of cohesin‐STAG2 in cancer. Oncogene 44, 277–287. 39613934 10.1038/s41388-024-03221-y

[17] Haarhuis JHI , van der Weide RH , Blomen VA , Yáñez‐Cuna JO , Amendola M , van Ruiten MS , Krijger PHL , Teunissen H , Medema RH , van Steensel B *et al*. (2017) The Cohesin release factor WAPL restricts chromatin loop extension. Cell 169, 693–707. 28475897 10.1016/j.cell.2017.04.013 PMC5422210

[18] Wutz G , Ladurner R , St Hilaire BG , Stocsits RR , Nagasaka K , Pignard B , Sanborn A , Tang W , Várnai C , Ivanov MP *et al*. (2020) ESCO1 and CTCF enable formation of long chromatin loops by protecting cohesinSTAG1 from WAPL. elife 9, e52091. 32065581 10.7554/eLife.52091 PMC7054000

[19] Casa V , Moronta Gines M , Gade Gusmao E , Slotman JA , Zirkel A , Josipovic N , Oole E , van IJcken WFJ , Houtsmuller AB , Papantonis A *et al*. (2020) Redundant and specific roles of cohesin STAG subunits in chromatin looping and transcriptional control. Genome Res 30, 515–527. 32253279 10.1101/gr.253211.119 PMC7197483

[20] Cuadrado A , Giménez‐Llorente D , De Koninck M , Ruiz‐Torres M , Kojic A , Rodríguez‐Corsino M and Losada A (2022) Contribution of variant subunits and associated factors to genome‐wide distribution and dynamics of cohesin. Epigenetics Chromatin 15, 37. 36424654 10.1186/s13072-022-00469-0 PMC9686121

[21] Viny AD , Bowman RL , Liu Y , Lavallée V‐P , Eisman SE , Xiao W , Durham BH , Navitski A , Park J , Braunstein S *et al*. (2019) Cohesin members Stag1 and Stag2 display distinct roles in chromatin accessibility and topological control of HSC self‐renewal and differentiation. Cell Stem Cell 25, 682–696. 31495782 10.1016/j.stem.2019.08.003 PMC6842438

